# Photocatalytic CO_2_ Conversion Using Metal-Containing Coordination Polymers and Networks: Recent Developments in Material Design and Mechanistic Details

**DOI:** 10.3390/polym14142778

**Published:** 2022-07-07

**Authors:** Lea-Sophie Hornberger, Friederike Adams

**Affiliations:** 1 Chair of Macromolecular Materials and Fiber Chemistry, Institute of Polymer Chemistry, University of Stuttgart, Pfaffenwaldring 55, 70569 Stuttgart, Germany; lea-sophie.hornberger@ipoc.uni-stuttgart.de; 2 Center for Ophthalmology, University Eye Hospital Tübingen, Elfriede-Aulhorn-Strasse 7, 72076 Tübingen, Germany

**Keywords:** photocatalysis, CO_2_ conversion, carbon dioxide reduction, artificial photosynthesis, metal-containing polymers, metallopolymers, porous organic polymers, porous coordination polymers, metal-organic frameworks, metal-organic layers

## Abstract

International guidelines have progressively addressed global warming which is caused by the greenhouse effect. The greenhouse effect originates from the atmosphere’s gases which trap sunlight which, as a consequence, causes an increase in global surface temperature. Carbon dioxide is one of these greenhouse gases and is mainly produced by anthropogenic emissions. The urgency of removing atmospheric carbon dioxide from the atmosphere to reduce the greenhouse effect has initiated the development of methods to covert carbon dioxide into valuable products. One approach that was developed is the photocatalytic transformation of CO_2_. Photocatalysis addresses environmental issues by transferring CO_2_ into value added chemicals by mimicking the natural photosynthesis process. During this process, the photocatalytic system is excited by light energy. CO_2_ is adsorbed at the catalytic metal centers where it is subsequently reduced. To overcome several obstacles for achieving an efficient photocatalytic reduction process, the use of metal-containing polymers as photocatalysts for carbon dioxide reduction is highlighted in this review. The attention of this manuscript is directed towards recent advances in material design and mechanistic details of the process using different polymeric materials and photocatalysts.

## 1. Introduction

In 2015, the United Nations stated 17 Sustainable Development Goals to fight climate change. Sustainable Development Goal 13 contains the mission of climate action: Adapting to climate change and investing in low-carbon development [1]. The mission started by dramatically decreasing greenhouse gas (GHG) emissions. Carbon dioxide is one of the GHGs, evolving from natural processes, but mainly from artificial activities. The amount of CO_2_ in the atmosphere has notably risen from the beginning of the industrial era and is steadily increasing. In 1750, at the beginning of the industrial revolution, the CO_2_ concentration in the atmosphere was 277 parts per million (ppm) [2] and has expanded to up to over 400 ppm measured in May 2022 [3].

Four different approaches can lead to a reduction in CO_2_ in the atmosphere: (1) Improving energy efficiencies in general, (2) use of energy sources that have zero or lower carbon dioxide exhaust such as solar or wind resources, (3) capturing the atmospheric CO_2_ geologically or (4) converting CO_2_ into valuable materials [4]. Conversion of carbon dioxide faces a number of hurdles since CO_2_ is the final product of carbon combustion and is a relatively inert gas. The reduction potential of CO_2_ vs. the Normal Hydrogen Electrode (NHE) E^0^ is −1.9 V, showing that in an electrochemical process, conversion demands high energy caused by high overpotential for a reaction taking place [5]. To overcome this drawback, catalysts are used to enable the economic conversion of CO_2_ into fuels and chemicals. Transforming atmospheric CO_2_ into C1/C2 fuels and other valued chemicals, such as carbon monoxide, methanol, formic acid, or methane, is a sustainable strategy for global carbon balance [6,7,8,9,10].

This review focusses on metal-containing polymers as catalysts for photochemical CO_2_ conversion. Nowadays, metal-containing polymers, also known as metallopolymers, are at the forefront for discovering new and sustainable homogeneous, as well as heterogeneous, catalysts. The combination of metal and polymer moieties merges the advantage of both materials: Metal centers build up the functional core as redox-active species, whereas polymeric materials are able to optimize the catalyst performance while acting as stabilizing structures due to their feature of being tunable. Especially for carbon dioxide conversion, metal-containing polymers can support photocatalytic [11,12,13,14,15], electrocatalytic [16,17,18,19,20], photo-electrocatalytic [13,15,21,22,23] or thermochemical [24] reduction reactions.

A photocatalytic reduction system comprises different components, namely, a catalyst, a photosensitizer and an electron sacrificing agent. Photosensitizers (PS) offer the ability to absorb light in the visible region and form electron-hole pairs. Sacrificial agents (SA) work as electron donors by reducing the photosensitizer [25,26]. This process scales down the recombination of electron and holes. That means that the redox potentials of the sacrificial agents and the photosensitizer have to be in accordance with each other. The photosensitizer is the species in the system that absorbs most of the light. The longevity of the excited state of the PS is required to be high enough to be reduced by the sacrificial agent. Therefore, organometallic photosensitizers consisting of metal atoms as the center combined with organic ligands, such as [Ru(bpy)_3_]^2+^ with 2,2′-bipyridine (bpy) ligands, were used. Water was mostly utilized as a sacrificial hole scavenger and solvent. However, oxidation from water to O_2_ required four electron holes (Table 1) and the generated O_2_ could act as an oxidizing agent having an adverse effect on the reduction reaction [27]. Therefore, organic substances were used as additional sacrificial agents, i.e., triethanolamine (TEOA), triethylamine (NEt_3_ or TEA), 1-benzyl-1,4-dihydronicotinamide (BNAH) or 1,3-dimethyl-2-phenyl-2,3-dihydro-1H-benzo[d]imidazole (BIH) [28].

**Scheme 1:**
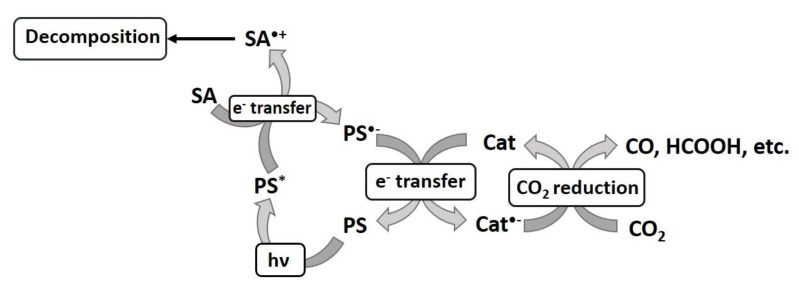
Basic mechanism for the photocatalytic CO_2_ reduction including sacrificial agent (SA), photosensitizer (PS) and catalyst (Cat). Data from Refs. [25,26].

After reduction by the SA, the photosensitizer can transfer the electron to the catalyst and return to the ground state. The metal center must be an electron-rich metal atom with filled d-orbitals as found in second- or third-row metals such as ruthenium or rhodium [25]. Organic ligands provide π-electron-accepting units for metal-to-ligand charge transfer (MLCT). The strong spin-orbit coupling in the second- or third-row metals enabled a pathway for excited electrons that switch multiplicities through intersystem crossing (ISC) [29,30]. Besides receiving electrons from the photosensitizer, the catalytic species needs to accumulate the electrons for facilitating the multi-electron reduction of CO_2_. For this purpose, CO_2_ needs to be adsorbed on the catalytic center. Metalorganic complexes are used with Mn(I), Re(I), Ru(II), Co(II), Ni(II), Ir(III) and Fe(III) transition metal centers that can coordinate carbon dioxide [25]. The surface adsorption activates the CO_2_ molecules and forms partially charged CO_2_^δ•−^ species. CO_2_ adsorption occurs via oxygen coordination, carbon coordination or mixed coordination. Either the oxygen atoms coordinate with their electron lone pairs by donating them to Lewis acid centers on the surface, the carbon accepts electrons from a Lewis base, or a mixed coordination of both processes happens. Adsorbed CO_2_^δ•−^molecules are no longer linear and have a bended structure [31]. After the catalyst assembles the electrons, these are transferred to CO_2_, which is reduced to give the reduction products, such as carbon monoxide, formic acid or methane (Scheme 1).

The whole process can be optimized by tuning the band energy of the light absorbing molecule. The light energy needs to be higher than its band gap to provide the required energy. Therefore, the photons of sunlight are divided up according to their energies/wavelengths into photons in the ultraviolet light range (λ < 400 nm, *E*_photon_ > 3.1 eV), visible light range (λ = 400–700 nm, 1.7 ≤ *E*_photon_ ≤ 3.1 eV), and infrared light range (λ > 400 nm, *E*_photon_ < 1.7 eV). The sunlight consists of 95% visible and infrared irradiation [32]. To be able to absorb light in this range, the band gap of the photocatalytically active materials is designed to be smaller than 3.1 eV. The band gap can not only be tuned for favorable light absorption but can also be adjusted for suitable potentials for subsequent redox reactions. The conductive band (CB) of the photocatalyst needs to be raised to a level which is higher than the reduction potential of CO_2_, while the valence band (VB) must exceed the oxidation potential of the electron donor in energy [14]. The reactions to the different products have distinct potentials. Table 1 presents the half-cell reactions and their appropriate potentials vs. the normal hydrogen electrode (NHE) at pH = 7 (*E*^0^’) [33,34].

In 1982, Lehn and Ziessel published one of the first innovative studies on the photochemical generation of carbon monoxide and hydrogen from CO_2_ and H_2_O. The authors used Ru(bipyridine)_3_^2+^ as the photosensitizer, Co^2+^ as the reducing intermediate/catalyst, and NEt_3_ as the electron donor dissolved in an aqueous acetonitrile solution. The addition of bipyridine affected the amount and ratio of generated gas. Lehn and Ziessel proposed an intermediate formation of Co(I) species in a reduction system together with [Ru(bpy)_3_]^2+^, but its nature was unknown at that point. It was only known that Co acted as a binder as well as an electrocatalyst for CO_2_ reduction [34]. Since then, interest in systems for CO_2_ photoreduction reaction has continuously grown due to their advantages over other methods: (1) The whole process is controllable and can be carried out under mild conditions in matters of reaction temperature and pressure; (2) when catalysts are completely recyclable, the overall consumption of all substances can be minimized, resulting in solely small molecule and atmospheric CO_2_ consumption; (3) the light source can be chosen individually by applying, e.g., renewable resources such as solar energy, and (4) the systems are modular and can easily be scaled-up [4,35,36].

**Figure 1:**
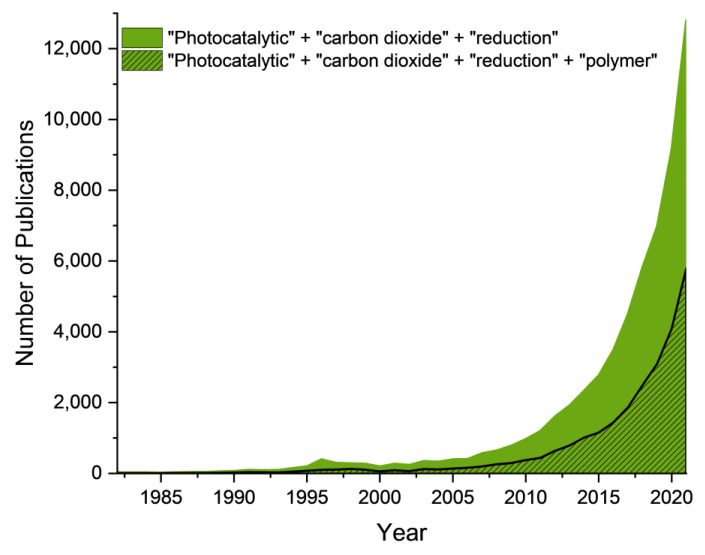
Publications per year between 1982 and 2021 that include the words “photocatalytic”, “carbon dioxide”, “reduction”, and “polymers”. Source: Google Scholar (Status: May 2022).

However, the mechanistic details for photocatalytic CO_2_ reduction including photosensitizer and sacrificing agents in the mechanistic circles are complex and mechanistic investigations are still ongoing. On the other hand, research on material design and efficiencies is simultaneously continuing to grow. The number of publications on photocatalytic carbon dioxide reduction exponentially increased up to 12,800 publications in the year 2021 alone (Figure 1). Additionally, publications on photocatalytic carbon dioxide reduction including polymers exponentially increased to 5790 in 2021, showing the high potential of polymers for assisting carbon dioxide conversion.

Challenges have emerged for photocatalysts in their performance due to low CO_2_ adsorptions, conversion efficiencies, and selective product generation [11,36,37,38]. Additionally, most of the photocatalytic systems lack stability, as it was revealed in several studies that a catalyst can barely be used for several catalytic cycles [11,36]. Stability issues caused by charges also influence the efficiency, showing that electrons and holes have to be separated adequately within the material to enhance stability. Moreover, the light absorption range is often limited and efforts need to be made to adjust this range to be in the visible light region. To sum up the requirements for photocatalytic systems: (1) suitable band gaps, (2) light absorption abilities in a broad range, (3) the efficient charge separation of electrons and holes, and (4) several adsorptive as well as reactive sites.

Recent advances have been made to overcome these obstacles such as new tailor-made materials combining photocatalysts and polymeric materials, which are summarized in this review article. As the number of materials for CO_2_ reduction is enormous, this manuscript focusses on recent developments in metal-containing polymeric systems. Different classes of 1D-coordination polymers and 2D-/3D-coordination networks, including supramolecular and coordination polymers, porous organic frameworks and metal-organic frameworks (MOFs) as photocatalysts, in addition to further strategies, are presented. Despite ongoing discussion regarding whether metal-organic frameworks can be considered as 3D-coordination polymers [39], we herein define MOFs and covalent organic frameworks (COFs) as coordination networks, a sub-category of coordination polymers, as also recommended by IUPAC [40].

## 2. 1D-Coordination Polymers

### 2.1. Re/Ru-Systems

In 2018, Rieger et al. published a micellar support for Re-metal complexes for CO_2_ reduction. They were the first to use an end group-functionalized polymer for the selective complexation of a rhenium complex. The authors synthesized a block copolymer via yttrium-mediated group-transfer polymerization (GTP) using hydrophobic 2-vinylpyridine (2VP) and hydrophilic diethyl vinylphosphonate (PDEVP) as monomers. AB-block copolymers (A = poly(2-vinylpyridine) (P2VP), B = poly(diethyl vinylphosphonate) (PDEVP)) were end-group functionalized with *ortho*-methylated bipyridines which were introduced as initiators of the yttrium catalysts. The photosensitizer and photocatalyst [Re(CO)_3_(bpy)Cl] was attached to the end-groups by complexation to yield Re-bpy-P2VP-PDEVP-block copolymers. Dynamic light scattering showed the ability of the polymers to self-assemble to micelles with tunable sizes dependent on the polymer chain lengths while protecting the Re-metal center in the micellar core. The systems were tested as photocatalysts for CO_2_ reduction in *N*,*N*-dimethylformamide (DMF) with TEOA as the sacrificial electron donor under LED light irradiation. In comparison to the respective molecular catalyst, the complexation to a P2VP-PDEVP-block copolymer structure increased the catalytic performance, producing CO as the exclusive product with a turnover number (TON) of 13 containing a 0.1 mM catalyst. The pure catalyst itself showed a TON of 6.5 in the same setup. It was proven that the attachment of a metal complex did not limit the catalytic properties and even enhanced them, resulting in higher TONs [41].

**Figure 2:**
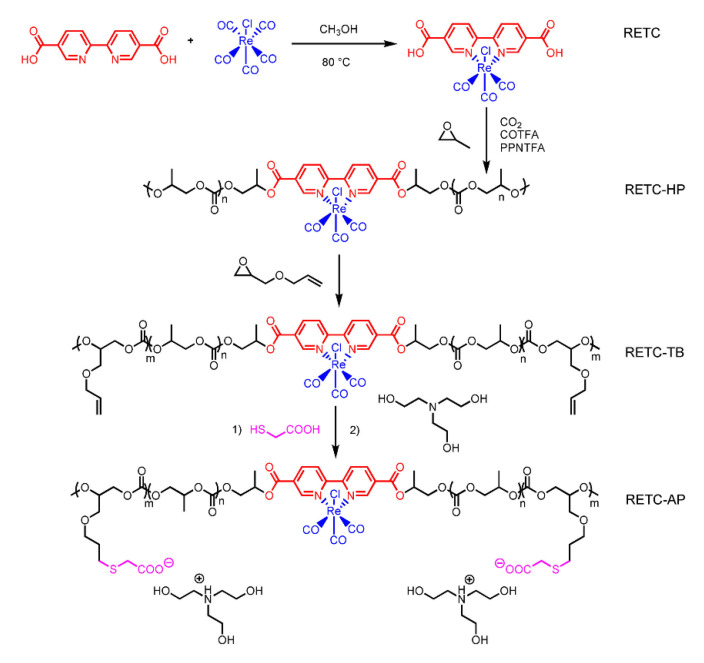
Synthesis of Re(dcbpy)-based amphiphilic micellar catalysts, starting from Re(dcbpy)(CO)_3_Cl (RETC) via Re-hydrophobic polymer (RETC-HP) and triblock Re-polymer (RETC-TB) resulting in Re-amphiphilic polymer (RETC-AP). Reprinted with permission from Ref. [42]. Copyright 2022, Wiley-VCH.

Since water is the most abundant and ecological solvent, in 2022, He et al. introduced the first visible light-induced material for photocatalytic CO_2_ reduction in aqueous media without the need for the addition of any further photosensitizer. The authors used triblock amphiphilic micelles which are able to self-assemble in water. The polycarbonate-based triblock polymeric micelles contained a rhenium complex in the backbone, namely Re(dcbpy) (dcbpy = 2,2′-bipyridine-5,5′-dicarboxylic acid), as a photocatalyst [42]. The material was synthesized by the copolymerization of CO_2_ and epoxide, followed by subsequent thiol-ene click reactions according to a previous publication [43]. In detail, tris-carbonyl-bromo(2,2′-bipyridine-5,5′-dicarboxylic acid) rhenium(I) (RETC) was synthesized by mixing [Re(CO)_5_Cl] and the bipyridine compound H_2_BPYDC, followed by the synthesis of the Re-hydrophobic polymer (RETC-HP) with bifunctional RETC as a chain transfer agent and propylene oxide (PO) as a monomer unit (Figure 2). Triblock Re-polymers (RETC-TB) were produced by using allyl glycidyl ether (AGE) as the second block. Thiol-ene click reaction of the RETC-TB with thioglycolic acid resulted in an amphiphilic Re-polymer (RETC-AP). Transmission electron microscopy (TEM) measurements of RETC-AP displayed spherical micelles with uniform particle sizes in water. The micelles had a core–shell structure with the hydrophilic polymer blocks in the outer sphere. The Re-functionality was protected in the hydrophobic core part. Polycarbonate chains suppressed dimerization of Re to maintain its activity. The functionality, as well as the morphology and porosity of the amphiphilic polycarbonate micellar rhenium catalysts, were easily regulated by the size of the hydrophilic and hydrophobic blocks. Photoreductions of CO_2_ were performed in water with TEOA as a sacrificial agent. Only CO and H_2_ were detected as products. CO selectivity was higher than 99% and the TON could be boosted to be 110, which is 37 times higher compared to the molecular Re catalyst in the organic solvent [42].

**Figure 3:**
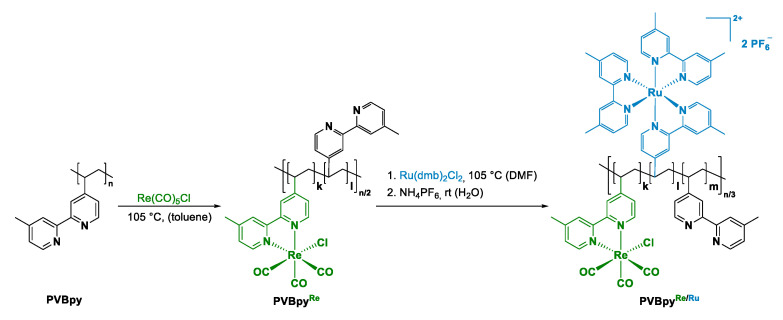
Two-step synthesis of the PVBpy^Re/Ru^ photocatalyst by loading with Re(CO)_5_Cl and Ru(dmb)_2_Cl_2_ [44].

The newest approach by Rieger et al. took advantage of Lewis pair mediated GTP with different Lewis acidic trialkyl aluminum compounds and Lewis basic phosphines to result in well-defined polymers with bipyridine monomer units for Re- and Ru-complexation. 4-Vinyl-4′-methyl-2,2′-bipyridine (VBpy) was catalytically polymerized for the first time and a combination of Al(*i*-BU)_3_ and PMe_3_ was determined as the optimum combination as a catalyst for polymerization. A two-step synthesis was performed to load the bipyridine bearing polymers, first with [Re(CO)_5_Cl] as the catalytic centers, followed by Ru(dmb)_2_Cl_2_ (dmb = 4,4′-dimethyl-2,2′-bipyridine) performing as a photosensitizer to give macromolecular rhenium-ruthenium complexes (Figure 3). In comparison to earlier studies, multiple bipyridine units were available in the polymer and not solely the end-group/initiating species was utilized. Polymer-complex structures were characterized by ultraviolet–visible (UV-vis), photoluminescence (PL) and infrared (IR) spectroscopy, as well as by inductively coupled plasma mass spectrometry (ICP-MS). The photocatalytic activity was measured under visible light irradiation in DMF with the addition of TEOA as the base and BIH as the sacrificial electron donor. Attachment of the rhenium and ruthenium centers to the poly(vinyl bipyridine) (PVBpy) offered proximity to these metal centers for an enhanced intramolecular electron transfer. PVBpy with a photocatalyst-to-photosensitizer ratio of 5% Re(I) compared to 95% Ru(II) showed a TON of 6088 and a turnover frequency (TOF) of 66 h^−1^ for the conversion of CO_2_ to CO due to effective electron transfers from the one electron reduced Ru(II) to the Re(I) centers [44].

**Figure 4:**
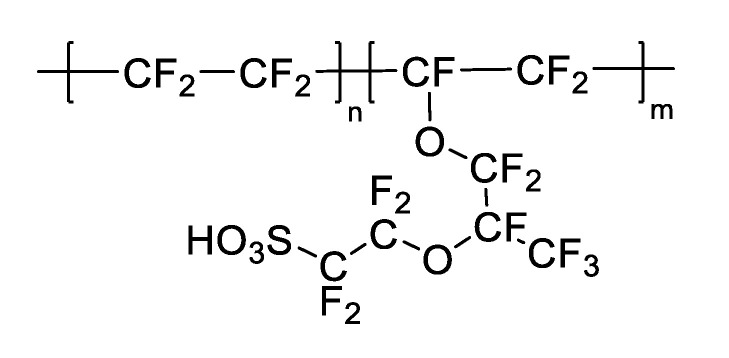
Structure of a Nafion ionomer.

Kim and Choi reported a molecular photosensitizer-catalyst assembly without the need of any chemical linkage, since linking the photosensitizer and the catalyst could require complex synthesis procedures. It can also cause disadvantages due to the close interactions of the functional centers on the one hand but prevents catalyst leaching on the other hand. Therefore, the authors utilized a Nafion polymer backbone (Nf) as a platform for attaching the photosensitizer and catalyst by electrostatic attraction [45]. Nafion ionomers were synthesized by the copolymerization of a perfluorinated vinyl ether comonomer with tetrafluoroethylene (Figure 4) [46]. The light absorber [Ru(bpy)_3_]^2+^ (RuL) and the catalyst *fac-*Re(bpy)(CO)_3_Cl:Re(I) (Re(I)) were both physically assembled on the Nafion platform in DMF. Re(I) was attached via hydrophobic interactions, as shown by infrared spectroscopy in the solid state. Washing with DMF proved only a weak bonding by physical interactions because Re(I) was easily washed away. RuL was linked in a stronger way via ionic interactions between the cationic RuL and the anionic sulfonic Nf group to form RuL-Nf. The binding was further characterized by monitoring the absorbance. UV-vis spectra of RuL-Nf in DMF showed that the Nf had no influence on the absorption characteristics of RuL. Photocatalytic reduction in carbon dioxide with Re(I)-RuL-Nf was tested in DMF with TEOA as the electron donor. CO production was analyzed by gas chromatography. RuL showed enhanced deactivation with higher RuL concentrations, indicating a self-sensitizing destruction. Upon excitation, the excited RuL* either reacted with the electron donor, or with itself by a self-destructive process. The role of the Nf was suggested to hinder this self-destruction and thus maintain the photoactivity and durability of the RuL photosensitizer. After reaction with TEOA, the excited and reduced RuL transferred its electron to the Re(I) followed by CO_2_ reduction to CO. The TON of Re(I)-RuL-Nf was calculated to be up to 454 after 10 h. This value was four times higher than the TON of 110 when using the molecular Re(I)-RuL catalytic system without Nafion fixation, proving the increased efficiency when using a Nafion backbone. Additionally, the presence of water dramatically decreased the photocatalytic reduction implying that water is not needed as a proton donor [45].

Another approach was provided by Marinescu et al. in 2019. The authors modified different supporting electrode materials with surface-immobilized conjugated polymers by electropolymerization. For this purpose, a [2,2′-bipyridine]5,5′-bis(diazonium) rhenium complex was dissolved in acetonitrile with additional 0.1 M tetrabutylammonium hexafluorophosphate as a conducting salt. Clean electrodes (TiO_2_ electrodes) were used as working electrodes. Electrochemical grafting was performed by cyclic voltammetry (CV) in several cycles. An increase in current measured by CV proved successful electropolymerization and an increasing amount of redox-active rhenium-bipyridine species available on the electrode surface. Film thickness grew with the number of CV cycles and, thus, with the number of catalyst-bipyridine species. The resulting orange films of poly(Re(CO)_3_Cl[2,2′-bipyridine]-5,5′diyl) were characterized by scanning electron microscopy (SEM) and atomic force microscopy (AFM) among other techniques, showing a uniform and smooth film growth across the chosen substrates. Photocatalysis studies of the conjugated polymers on mesoporous TiO_2_ substrates were conducted in DMF with TEOA as sacrificial agent. While the pure TiO_2_ substrates only generated 0.11 μmol CO in 5 h, the substrate with the lowest amount of conjugated polymer, electropolymerized with using solely one CV scan, showed a TON of 70 and a TOF of 14 h^−1^ after 5 h of irradiation. Using a substrate with higher catalyst loading, produced by five CV cycles, increased the TON to up to 31 with a TOF of 6.1 h^−1^. Films with even higher catalyst loadings did not exceed these numbers, which indicated that only small quantities of deposited active material remained active over the 5 h of irradiation [47].

### 2.2. Ni, Fe, Mn-Systems

Cheaper and more abundant non-noble metals were also tested, as their potential for practical applications is greater due to their lower prices, greater availability, and lower toxicities. Very recently, Wang, Wang and Cao used another technique for immobilizing catalysts on semiconductors for an enhanced charge separation. They published a hybrid assembly with Nickel poly-pyridine polymers (NiP) binding on CdS quantum dots (QDs) via thiophene immobilization. NiP was synthesized by polymerizing 2-vinylthiophene and nickel poly-pyridine complex (NIL) mediated by azobisisobutyronitrile (AIBN) [48]. MPA-CdS QDs (MPA = 3-mercaptopropionic acid) were prepared according to another publication [49]. Since NiP contained thiophene units, mixing NiP and MPA-CdS QDs resulted in CdS-Ni assemblies due to the binding affinity between sulfur and cadmium The assembly was investigated by high-resolution TEM (HR-TEM), X-ray photoelectron spectroscopy (XPS), Fourier-transform infrared spectroscopy (FT-IR), and ICP-MS. Photocatalytic activity in terms of CO_2_ reduction was tested in a mixture of water and TEOA as the electron donor. The new material assembly provided syngas production with 5500 μmol g^−1^ h^−1^ under visible light irradiation. Syngas is an important feedstock for the chemical industry, consisting primarily of hydrogen and carbon monoxide as it is industrially used, e.g., for Fischer-Tropsch processes. The syngas composition could be controlled upon changing the ratios of MPA-CdS QDs and NiP during the assembly. With a 1:1 ratio of the two material components, the H_2_/CO ratio was observed to be around 4:1. The H_2_/CO ration was adjustable from 4:1 to 1:3 with lower NiP concentration compared to CdS cores indicating that the nickel center was more sensitive to H_2_ production, whereas higher CdS amounts seemed to yield more CO [48].

## 3. 2D- or 3D-Coordination Networks

### 3.1. Supramolecular Polymers and Polymer Gels

**Figure 5:**
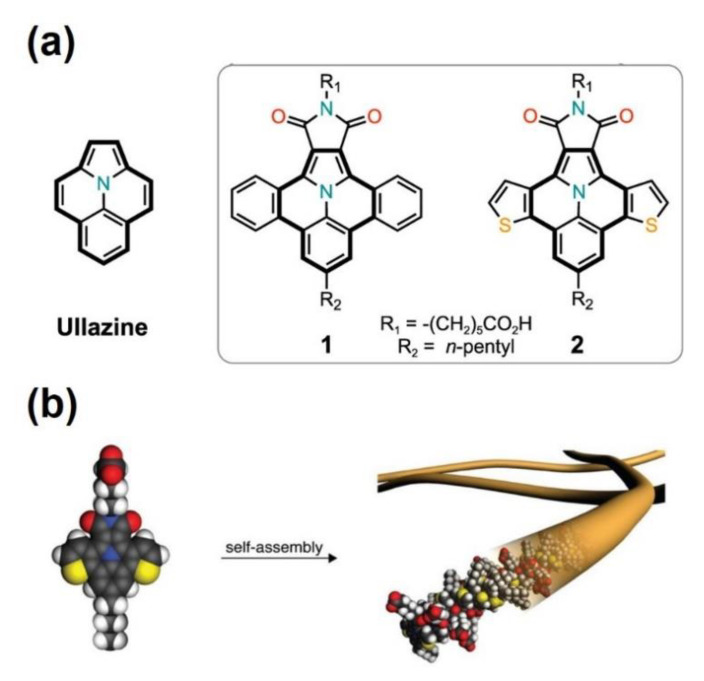
(**a**) Chemical structures of ullazine and diareno-fused ullazines with two benzo (1) and two thieno rings (2); (**b**) Molecular graphics of ullazine (2) and its self-assembly into a supramolecular fiber. Adapted with permission from Ref. [52]. Copyright 2022, American Chemical Society.

Another strategy to design photosynthetic systems are self-assembled supramolecular polymers. This class of material opens the possibility for customizable systems with tailored functionalities. Covalently connected polymer gels enable the co-location of, e.g., a photosensitizer and a catalyst, as nature presents in its photosynthesis and energy-conversion devices. The covalent bonding of functional groups for photocatalytic reduction within the artificial supramolecular systems increases the efficiency of electron transfer, leading to an enhancement of chemical transformations [50]. Moreover, certain structures are able to undergo π-stacking. Previous research has shown that π-π stacked supramolecular polymers are accompanied by enhanced charge separation [51]. Stupp et al. specialized in synthesizing self-assembled supramolecular polymers that can feature both: photosensitize CO_2_ reduction reactions while remaining stable in aqueous reaction media. Their strategy included using supramolecular polymers containing chromophores for light absorption and a catalyst that can promote CO_2_ reduction [52]. Amphiphilic structures are the prerequisite for the self-assembly of tailored structures in an aqueous environment [53]. Therefore, amphiphilic chromophores with diareno-fused ullazine cores were used as monomers. 1,3-dipolar cycloaddition of azomethine ylide with dipolarophile *tert*-butyl 6-maleimidohexanoate was used for the synthesis of compounds 1 and 2 (Figure 5). For generating amphiphilicity, monomers bearing monoimide groups containing carboxylic acids as hydrophilic head group were introduced. *n-*Pentyl tails served as hydrophobic units. The extension of the ullazine core and its π-system by two benzo (1) or two thieno rings (2) improved the absorption ability for visible light, as proven by UV-vis absorption and fluorescence spectroscopy. Aqueous self-assembly was enabled by dissolving the free acids in water using equimolar amounts of NaOH, followed by the addition of NaCl. Self-assembled in water, the monomers built entangled fibers on a nanoscale, as proven by cryogenic transmission electron microscopy (cryo-TEM) resulting in hydrogel formation. The hydrogels were further coupled with a binuclear cobalt catalyst. Photocatalytic CO_2_ reduction was first performed in acetonitrile (MeCN) and water mixtures, but also in pure water, while all experiments contained TEOA as a sacrificing electron donor. CH_4_ and CO were produced with turnover numbers of TON_CO_ = 1136 and TON_CH4_ = 490 using the self-assembled cobalt-nanofibers (1) with benzo rings, and TON_CO_ = 1525 and TON_CH4_ = 865 for the other system, and (2) with thieno rings under blue 450 nm light irradiation for 48 h. The photocatalytic activity of fibers (2) was also shown to be stable over 6 days in a fully aqueous medium with turn over numbers of 4625 and 1518 for CO and CH_4_, respectively. Mechanistic studies revealed an oxidative electron transfer from the sacrificing agent TEOA to the sensitizing hydrogel, which donated electrons to the cobalt-active sites for CO_2_ reduction, resulting in CO and CH_4_ production [52].

**Figure 6:**
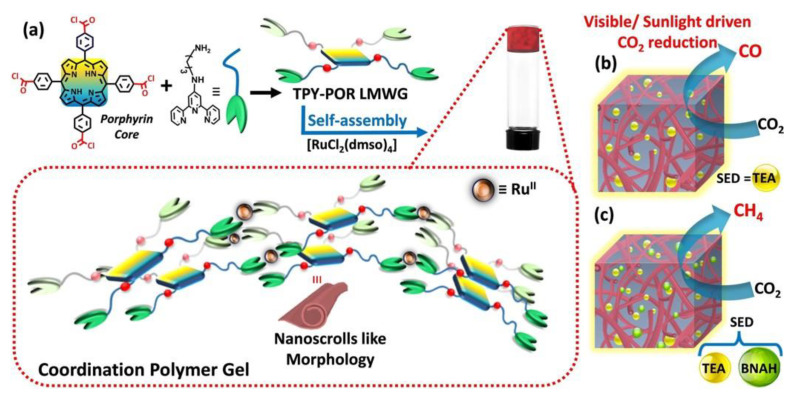
(**a**) Synthesis and self-assembly of TPY-POR LMWG monomers with [RuCl_2_(dmso)_4_] (dmso = dimethyl sulfoxide) to form a coordination polymer gel; (**b**) Visible or sunlight driven CO_2_ reduction with TEA as single electron donor (SED); (**c**) Visible or sunlight driven CO_2_ reduction with TEA and BNAH as SED. Reprinted with permission from Ref. [54]. Copyright 2022, Wiley-VCH.

Recently, Maji et al. reported on a metal-organic coordination polymer gel as a material for carbon dioxide reduction. As the low molecular weight gelator (LMWG), the authors used a porphyrin core connected to four terpyridine units (TPY-POR). It was synthesized by an amide coupling reaction of 4,4′,4″,4‴-(Porphine-5,10,15,20-tetrayl)tetrakis benzoic acid with 2,2′:6′,2″-terpyridine-4′-yl-propane-1,3-diamine (Figure 6). While the four amide units acted as H-bonding sites, the terpyridine units were used to bind four metal complexes. Moreover, the terpyridine moieties served as the linkers, improving exciton mobility and overall charge mobility. Self-assembly was proven by HR-TEM and powder X-ray diffraction (XRD) studies. The porphyrin-based monomers self-assembled together with Ru(II) ions to a fully coordinated polymer gel (Ru-TPY-POR CPG) (Figure 6a) [54]. Ru(II) was chosen due to its well-studied photocatalytic activity [55]. The self-assembly was promoted by intermolecular π-π stacking and H-bond interaction between the chromophores. The nanoscopic material combined a chromophore acting as a photosensitizer and a catalyst and thus enabled charge transport within the entire network. This novel metal-organic gel showed impressive conversion of CO_2_ to CO in the presence of triethylamine (TEA) sacrificial electron donor under visible light irradiation in an acetonitrile/water mixture. A high production rate of 3500 μmol g^−1^ h^−1^ with a selectivity of over 99% over CH_4_ was observed when using TEA as SA (Figure 6b). Furthermore, the catalyst was stable without losing its performance for more than six cycles [54]. Previous research showed that the addition of BNAH was beneficial for photoreduction due to lower oxidation potential in comparison to TEA [56]. In this study, the addition of BNAH along with TEA facilitated the formation of CH_4_ with a selectivity of 95% over CO, with a high production rate of 6700 μmol g^−1^ h^−1^. CH_4_ was more difficult to produce as it required an 8e^−^/8H^+^ reduction process but quenching of BNAH enabled this complex reduction (Figure 6c). All photocatalytic reactions, with and without the addition of BNAH, were also proved to happen equally under natural sunlight irradiation instead with the use of an artificial Xe lamp providing visible light. Mechanistic investigations included in situ diffuse reflectance infrared Fourier-transform spectroscopy measurements and quantum chemical calculations for computing Gibbs free energies. It was revealed that the porphyrin units acted as a photosensitizer and [Ru(TPY)_2_]^2+^ as the catalytic centers for CO_2_ reduction. The first mechanistic step was the photoexcitation of porphyrin accompanied by one electron transferred to [Ru(TPY)_2_]^2+^, resulting in [Ru^II^(TPY^•−^)(TPY)]^+^. The intermediate was then reduced to [Ru^I^(TPY^•−^)(TPY)]. One pyridine nitrogen was substituted by CO_2_ to form [Ru^II^(TPY)(*η*^2^-TPY)(COO^2−^)]. The next protonation step was shown to be thermodynamically favored resulting in [Ru^II^(TPY)(*η*^2^-TPY)(COOH^−^)]^+^. Further protonation was accompanied by the elimination of a water molecule to result in [Ru^II^(TPY)(η^2^-TPY)(CO)]^2+^. Reduction and desorption of CO enabled the regeneration of [Ru^II^(TPY^•−^)(TPY)]^+^. This regeneration step could not be shown in the presence of BNAH. When BNAH was oxidized, deprotonation and further dimerization to BNA_2_ was induced [54]. The reduction potential of the BNA_2_ dimer exceeded the one of BNAH, facilitating the quenching of the photosensitizer [56]. [Ru^II^(TPY^•−^)(*η*^2^-TPY)(CO)]^+^ was protonated by BNAH to [Ru^II^(TPY^•−^)(*η*^2^-TPY)(COH)]^+^. The higher reduction potential of BNAH enabled further reduction and protonation to [Ru^II^(TPY^•−^)(*η*^2^-TPY)(CH_2_O)]^+^, [Ru^II^(TPY^•−^)(*η*^2^-TPY)(OCH_3_)]^+^, and [Ru^II^(TPY^•−^)(*η*^2^-TPY)(HOCH_3_)]^+^. Protonation and simultaneous reduction promoted water elimination to produce [Ru^II^(TPY^•−^)(η^2^-TPY)(CH_3_)]^+^. Final proton-coupled electron transfer released CH_4_ and [Ru^II^(TPY^•−^)(TPY)]^+^ was regenerated. These mechanistic insights revealed that covalent linkage of the photosensitizer and the catalyst could be a key factor for high performances and the choice of SA is critical for product selectivity [54].

In 2020, Sun et al. presented the first pyridyl-salen-based ligands (H_2_L) with metal-containing (Mn(III) or Fe(III)) coordination polymers (CPs) that can build supramolecular structures with hydrogen bonds. The ligand combined a pyridine moiety with salen ligands containing N_2_O_2_ coordination pockets [57]. Pyridine moieties are known to be stable catalysts for aqueous electrochemical reductions in multiple-electrons and multiple-protons [58,59,60] but lack light absorption. Salen moieties have attractive light absorption properties and electron transfer ability together with the CPs. No matter which metal center was used (Mn(III) or Fe(III)), the coordination polymers showed high photocatalytic activity, as measured by electrochemical impedance spectroscopy (EIS). Nevertheless, Fe(III) materials had a slightly faster interfacial charge transfer and, thus, a better performance than Mn(III). EPR studies helped to unravel the reaction mechanism of the pyridyl-salen coordination polymer system paired with TEOA hydrogen and electron donor. These results implied that the ligand center acted as the catalytic active site and not the Fe(III)/Mn(III) metal center in the reduction from CO_2_ to CO. CO was observed as the only product in the gas phase. It was produced by the Fe(III) CP with 31.6 μmol g^−1^ h^−1^, by Mn(III) CP with 14.3 μmol g^−1^ h^−1^, and by the single ligand with 4 μmol g^−1^ h^−1^. After light absorption and reduction by TEOA, the ligand radical of H_2_L was formed, reducing CO_2_. The superior catalytic activity of the porous organic polymer together with Fe(III) coordination could be explained by improved light absorption, as well as the enhanced separation of charge carriers [57].

**Figure 7:**
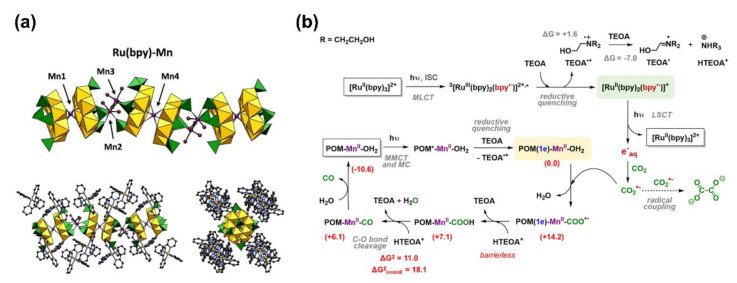
(**a**) Generation of chains by connected M(P_4_Mo_6_) sandwich-type POMs with Mn(II) ions, side (left) and top view (right); (**b**) Schematic presentation of the mechanism of the photocatalytic reduction of CO_2_ to CO with [Ru(bpy)_3_]^2+^ as a photosensitizer and TEOA as sacrificial agent using a POM system bearing Mn(II). Gibbs free energies (ΔG) are given in kcal mol^−1^. MLCT, ISC, MMCT, MC, and LSCT are abbreviations for metal-to-ligand charge transfer, intersystem crossing, metal-to-metal charge transfer, metal-centered, and ligand-to-solvent charge transfer, respectively. Adapted with permission from Ref. [61]. Copyright 2021, American Chemical Society.

Recently published in 2022, Mellot-Draznieks and Dolbecq revealed insights into the mechanism of photocatalytic CO_2_ reduction via density functional theory (DFT) calculations. Therefore, the groups synthesized three heterometallic molybdenum(V) phosphates containing polyoxometalate (POM) units under hydrothermal conditions. A metal ion of either Mn(II) or Co(II) was embedded between two P_4_Mo_6_^V^ anionic rings. For the generation of a completely inorganic photocatalyst, Fe(II) ions were mixed with the Mn(II)-containing reaction medium building a 3D network of Fe-Mn. Fe(II) and Fe(III) as well as Mn(II) centers connect the POM units in a network acting as counter ions to extra phosphate ions, that do not belong to P_4_Mo_6_ rings (Figure 7a). Besides the latter novel structure, additional structures were synthesized by [Ru(bpy)_3_]^2+^ complexes that were added to both Mn(II) and Co(II) POM unit solutions. The Ru bipyridine complexes served as counter ions to the anionic chains that were connected via Mn(II) and Co(II) ions to Ru(bpy)-Mn and Ru(bpy)-Co hybrids. Testing all three compounds for photoreduction, solely Fe-Mn and Ru(bpy)-Mn complexes were able to catalyze the reduction of CO_2_ to CH_4_ and CO as a side product. TEOA was used as a sacrificing agent and [Ru(bpy)_3_]^2+^ as a photosensitizer. DFT calculations of these bipyridine systems revealed new knowledge leading to a novel proposed mechanism, in which [Ru(bpy)_3_]^2+^ acted as a catalyst and the POM material as a co-catalyst (Figure 7b). The [Ru(bpy)_3_]^2+^ complex was reduced via photoionization in a one-electron process to yield the [Ru^II^(bpy)_2_(bpy^•−^)]^+^. One-electron charge transfer to the solvent (water) reconverted this structure to a [Ru(bpy)_3_]^2+^ species. The electron was solvated and could reduce CO_2_ to CO_2_^•−^ because of its tendency to localize in an empty π* molecular orbital of the carbon dioxide. The reduced CO_2_^•−^ coordinated to a metal(II) ion (e.g., Mn(II)) via its carbon atom by replacing a water ligand that was coordinated to the metal atom, forming a metal-COO^•−^ intermediate. This Mn-COO^•−^ intermediate was further reacted to Mn-COOH via electron transfer from a POM cluster and proton transfer from HTEOA^+^. At this point, reduced POM units, acting as a co-catalyst, and the sacrificing agent, which can generate protons via photooxidation, were both involved. Another suggestion was made that TEOA^•^ might act as a proton and electron donor concurrently. For C-O(H) cleavage, Mn-COOH was again protonated by a second HTEOA^+^. The author showed the necessity of a metal-containing photosystem because otherwise CO_2_^•−^ itself would dimerize to oxalate. Further suggestions were made that CO could probably be reduced to CH_4_ by the POM materials and TEOA without the influence of [Ru(bpy)_3_]^2+^. For the system containing Co(II)-POM units and [Ru(bpy)_3_]^2+^, DFT calculations could additionally show that these systems were unable to reduce CO_2_. The authors stated that Co(II) units dimerized while adsorbing an additional electron, making them inactive for CO_2_ reduction [61].

### 3.2. Porous Organic Polymers

Porous organic polymers (POPs) are macromolecules mainly composed of carbon, nitrogen, and oxygen atoms, in addition to other non-metallic chemical elements that are linked covalently. POPs possess high porosity with permanent pores due to their tailor-made structures. They can be classified by crystallinity to distinguish between crystalline covalent organic frameworks (COFs) and amorphous polymers. Amorphous polymers contain a wide variety of materials such as conjugated microporous polymers (CMPs), hyper-crosslinked polymers (HCPs), etc. [62]. Covalent triazine frameworks (CTFs) are another class of POPs that, based on current research, contain crystalline and amorphous materials. Their aromatic CN-linkage of the triazine unit, as well as the absence of weak bonds, gives them unique characteristics [63]. The porous structure makes the substrate accessible for small gas molecules and thus provides enhanced gas adsorption ability.

POPs in general are a class of versatile materials due to their broad light absorption range caused by the tunable positions of both: the valence and conduction band. They offer even more tunable characteristics such as surface area or the incorporation of different functionalities by adding various building blocks or active sites. The surface area is characterized by the Brunner-Emmet-Teller (BET) specific surface area. Besides the surface area, pore sizes as well as chemical structure are variable to tune the CO_2_ adsorption capability [62,64].

Thornton et al. introduced four general steps for the catalytic process of CO_2_ reduction with zeolite catalysts [65]. Zeolites are aluminosilicates in which the crystalline 3D structure is built from four-connected tetrahedral frameworks making them microporous minerals [66]. The zeolites’ photocatalytic mechanism can be adapted to POPs: (1) Carbon dioxide diffuses into the active sites of the material, (2) CO_2_ is adsorbed on the active catalytic sites, (3) carbon dioxide is converted into product species, and (4) the product molecules are desorbed and leave the material [65].

#### 3.2.1. Crystalline Frameworks

COFs offer an extended π-system due to the covalent organic framework and the supplementary crystallinity. The high conjugation degree enhances the transport and separation of photogenerated electrons and holes, which boosts charge separation for enhancing the lifespan of excited states [67].

**Figure 8:**
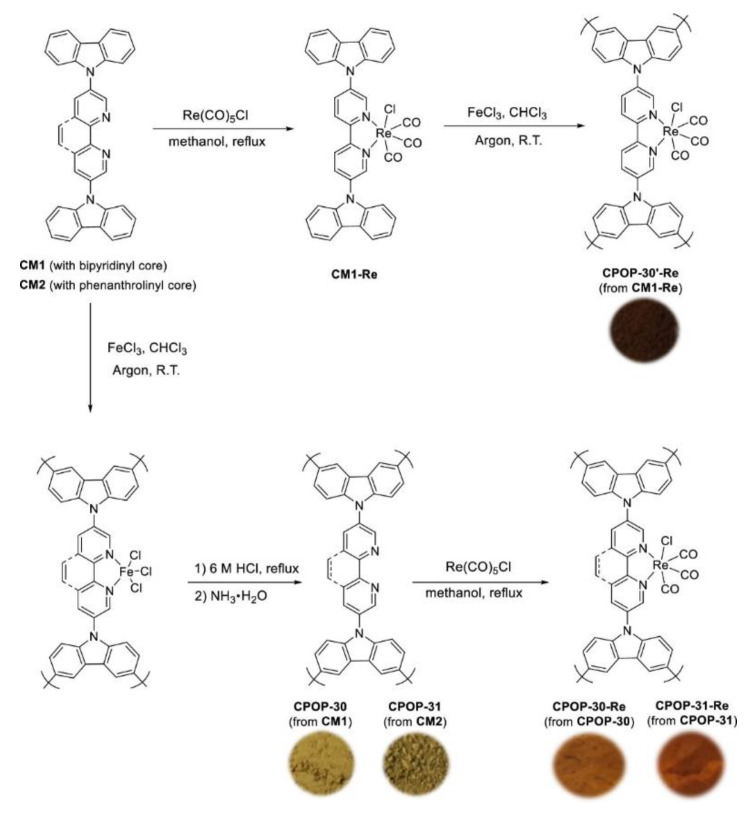
Direct polymerization of carbazole-based, rhenium-bearing monomers resulting in CPOP-30′-Re and synthesis of rhenium-metalated polypyridine-based porous polycarbazole networks (CPOPs) via post-synthetic metalation of CPOP-30 or CPOP-31. Reprinted with permission from Ref. [68]. Copyright 2019, American Chemical Society.

Noble metals, such as Re or Ru, have shown to be suitable components for photocatalytic CO_2_ conversion, especially when mixed with organic networks. The groups of Thomas and Han used FeCl_3_-promoted oxidative coupling polymerization of 5,5′-di(9*H*-carbazol-9-yl)-2,2′-bipyridine (CM1) or 3,8-di(9*H*-carbazol-9-yl)-1,10-phenanthroline (CM2) to produce porous polycarbazole networks and included catalytic rhenium(II) centers in their materials (Figure 8). The authors compared two materials: a bipyridine-based (CPOP-30) and a phenanthroline-based (CPOP-31) polycarbazole network. Both materials contained nitrogen-units for anchoring Re(I) active species, so that the polycarbazole networks could be metalated to CPOP-30-Re/CPOP-31-Re via the post-synthetic route by metalation of the polycarbazole. Direct polymerization of carbazole-rhenium monomers resulted in CPOP-30′-Re. All carbazole networks, CPOP-30-Re, CPOP-30’-Re and CPOP-31-Re, formed covalent organic frameworks (COFs) with a regular π-π stacked structure. The metal-loaded polymer networks were used as photocatalysts in organic solvents, with an additional sacrificial agent TEOA or TEA. CPOP-30′-Re lacked in performance as it only had a CO production of 3.2 3 μmol within four hours and 10 mg of catalyst. The pyridine-based COF CPOP-30-Re outperformed the phenanthroline-based CPOP-31-Re framework. The phenanthroline framework had a larger delocalization of charges and less negative reduction potential, weakening the thermodynamic driving forces and thus the performance in CO_2_ reduction. The authors found out that the frameworks themselves also performed as light-harvesting photosensitizers besides keeping chelating sites. With the pyridine-based framework, CO formation could be achieved with production rates up to 623 μmol g^−1^ h^−1^ in less than 10 h with a CO selectivity of almost 98% [68].

In a similar approach, Chen, Sprick, and Cooper used bipyridine units in an oligomer-linked framework for anchoring Re(I) complexes. The authors utilized Knoevenagel condensation reactions of 1,3,6,8-tetrakis(4-formylphenyl)pyrene (TFPPy) and 5,5′-bis(cyanomethyl)-2,20-bipyridine to obtain a fully π-conjugated backbone and thus an increased conjugation framework. Besides bipyridine for anchoring [Re(CO)_5_Cl] complexes via post-synthetic modification, the COF comprised cyanovinyl-groups due to the Knoevenagel reaction, which supported CO_2_ uptake. Transient absorption spectra revealed a long-lived charge separated state by the presence of Re within the COF. Computational calculations including DFT and time-dependent (TD) DFT calculations proved electron transfer upon electronic excitation from the COF backbone to the anchored Re-complexes. Photocatalytic reduction experiments were performed under visible light in acetonitrile, with TEOA as a sacrificial agent. CO production showed a selectivity of 81% and a rate of up to 1040 μmol g^−1^ h^−1^. With the addition of a supplementary photosensitizer 4,4′-*bis*(1,1-dimethylethyl)-2,2′-bipyridine-*N*1,*N*1′]*bis*[3,5-difluoro-2-[5-(trifluoromethyl)-2-pyridinyl-*N*]phenyl-C]iridium(III)-hexa-fluorophosphate (Ir[dF(CF_3_)ppy]_2_(dtbpy))PF_6_ (dF = difluoro, ppy = 2-phenylpyridine, dtbpy = 4,4′-di-tert-butyl-2,2′-dipyridyl)), embedded in the accessible pores of the COF, the CO production rate was maximized to 1400 μmol g^−1^ h^−1^ and a selectivity of 86% was reached by an electron transfer mechanism between the photosensitizer and COF by oxidative quenching. Moreover, colloidal Pt was added via in situ photodeposition. The formation of syngas was enhanced and the H_2_:CO ratio was influenced from 1:4 to up to 10:1 by adding different amounts of Pt between 0 and 4 wt.%. Higher amounts of Pt caused higher selectivity towards H_2_ [69].

**Figure 9:**
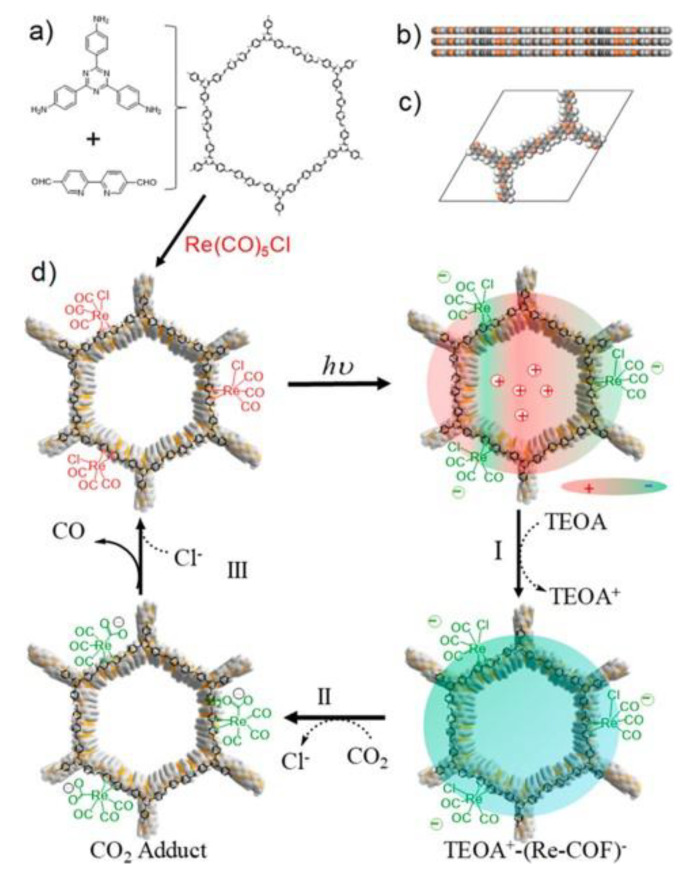
(**a**) Synthesis approach of the 2D COF; (**b**) Side view of AA stacking; (**c**) Unit cell of AA stacking COF; (**d**) Proposed mechanism of the photocatalytic CO_2_ reduction. Reprinted with permission from Ref. [70]. Copyright 2019, American Chemical Society.

Zhang and Huang designed a 2D-COF consisting of 2,2-bipyridyl-5,5-dialdehyde (BPDA) and 4,4′,4″-(1,3,5-triazine-2,4,6-triyl)trianiline (TTA) reacted via solvothermal reactions (Figure 9a). Postsynthetic incorporation of [Re(CO)_5_Cl] formed Re-COF systems. Single COFs stacked together in an AA stacking mode (Figure 9b,c) so that all layers showed identical lateral coordinates. These observations were supported by simulations using Material Studio 8.0 since this stacking was calculated to be the most probable state. In situ diffuse reflectance UV-vis spectroscopy helped to reveal mechanistic details (Figure 9d). Absorption of light induced an intramolecular charge transfer by the Re-COF structure, which was immediately quenched by TEOA, resulting in a TEOA^+^-(COF-Re)^−^ state with charge separation. Afterwards, CO_2_-Re complexes were formed after Cl^−^ dissociated from the Re precursor. Finally, CO was steadily generated, which was assigned as the rate limiting step of the reaction [70].

A novel approach in the field of photocatalysts for CO_2_ reduction was reported in 2017 by Inagaki and Ishida et al. They were the first to use periodic mesoporous organosilica (PMO) containing bipyridyl ligands in the pore walls. These bipyridyl units facilitated the coordination of two different metal complexes: a Ru photosensitizer (Ru(PS)) and a Ru catalyst (Ru(Cat)) by stepwise reaction to build a heterogeneous photocatalyst structure with the active sites being embedded inside the pores. Photochemical reduction in CO_2_ under visible light with the Ru(PS)-Ru(Cat)-Bpy-PMO system in aqueous dimethylamine (DMA) solution and BNAH as electron donor yielded CO and formate as products. Molar fractions of the photosensitizer and catalyst (*x*, *y*) were determined by XRD measurements. Moreover, the molar fraction of Ru(Cat) (*y*) was also quantified by the amounts of CO being dissociated from the Ru(Cat) under UV-light irradiation. The (*x*, *y*) ratio could influence the product selectivity. The CO selectivity increased by raising molar fractions of Ru(PS) to *x* = 0.11 and Ru(Cat) to *y* = 0.055, resulting in TONs over 162 h^−1^ and a CO selectivity of 47% vs. 37% for formate. The durability of the tested POMs was sufficient to be recycled and reused at least three times [71].

Cheaper and more abundant non-noble metals were also tested as photocatalysts in COF structures. In 2019, Lan et al. reported on COF structures built from covalently linked 5,10,15,20-tetrakis(4-aminophenyl)-porphinato (TAPP-M, M = 2H, Zn, Ni, Cu) and 2,3,6,7-tetra(4-formylphenyl)-tetrathiafulvalene by Schiff-base condensation resulting in (TTCOF-M). Testing CO_2_ reduction without using any sacrificial agent or co-catalyst, only TTCOF-Cu and TTCOF-Zn, showed significant photocatalytic activity. TTCOF-Zn showed the highest CO production, with values of 12.33 μmol after 60 h; TTCOF-Cu performed weaker and caused a CO production of 8.65 μmol. The authors proposed an intrinsic mechanism for their COFs with metalloporphyrin and tetrathiafulvalene moieties. After photoexcitation, the tetrathiafulvalene moieties functioning as HOMO centers transferred the electrons to the metalloporphyrins serving as LUMO centers. Afterwards, the electrons were transported to the catalytic metal centers in the TAPP units for CO_2_ reduction, while H_2_O was oxidized by tetrathiafulvalene gaining electrons for charge balance. No photosensitizer, sacrificial agents or co-catalyst were needed for a CO selectivity of almost 100% [72].

Wang et al. designed porphyrin-tetraphenylethene-based COFs (MP-TPE-COF) with anchored Ni(II) metals. The anchored Nickel caused a CO production rate of 525 μmol g^−1^ h^−1^ with 93% selectivity, as well as a high durability in aqueous solution. TEOA served as an electron donor and [Ru(bpy)_3_]Cl_2_ as a photosensitizer. Besides DFT calculations, mechanistic investigations included EIS and steady-state photoluminescence measurements: the photosensitizer was excited and reduced Ni(II) to give Ni^0^ which could adsorb and activate CO_2_. Protonation generated coordinated COOH species to the Ni(II) intermediate. Protonation and water release produced CO as a product and regenerated Ni(II) active sites [73].

**Figure 10:**
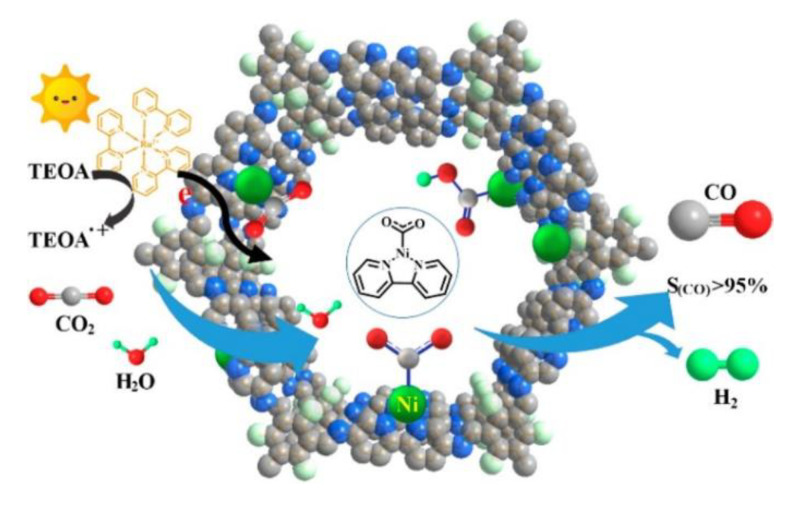
Schematic diagram of the photocatalytic CO_2_ reduction by a 2,2′-bipyridine-based COF with embedded Nickel sites. Reprinted with permission from Ref. [74]. Copyright 2019, American Chemical Society.

2,2′-bipyridine-based COFs with single Ni sites were reported by Yu and Zou in 2019. Condensation of 1,3,5-triformylphloroglucinol and 5,5′-diamino-2,2′-bipyridine under solvothermal conditions led to the 2,2′-bipyridine-based COF (TpBpy). Treatment of TpBpy with Ni(ClO_4_)_2_ inserted Ni ions to give Ni-TpBpy. FT-IR spectroscopy and ^13^C-NMR spectroscopy revealed the preservation of the COF structure after Ni loading and XPS measurements confirmed the successful Nickel loading. N_2_ adsorption and desorption measurements showed a decrease in the BET surface area from 973 to 580 m^2^ g^−1^, which further indicated the loading with Ni(II) ions in the COF structure by chelation of the bipyridine binding units. The photocatalytic activity in CO_2_ reduction was measured in aqueous acetonitrile, with TEOA as a sacrificing agent and a Ru photosensitizer. The system induced a CO production rate of 811 μmol g^−1^ h^−1^ with a selectivity of 96%. The experimental data and DFT calculations opened suggestions for a mechanism: the [Ru(bpy)_3_]^2+^ photosensitizer was first excited. Then, the excited electrons were transferred through the COF to the adsorbed CO_2_ on the Ni(II) sites. Higher CO_2_ affinity of the Ni sites in comparison to H^+^ adsorption determined the selectivity of the formed product in terms of inhibiting H_2_ formation. Compared to free CO_2_, adsorbed species had a bended configuration demonstrating its activation by metal sites (Figure 10). In general, the bipyridine moiety hosted the CO_2_ and metallic catalytic centers and additionally facilitated the activation of CO_2_. This combined functionality could be enabled by synergistic effects between the catalytic sites and the framework [74].

**Figure 11:**
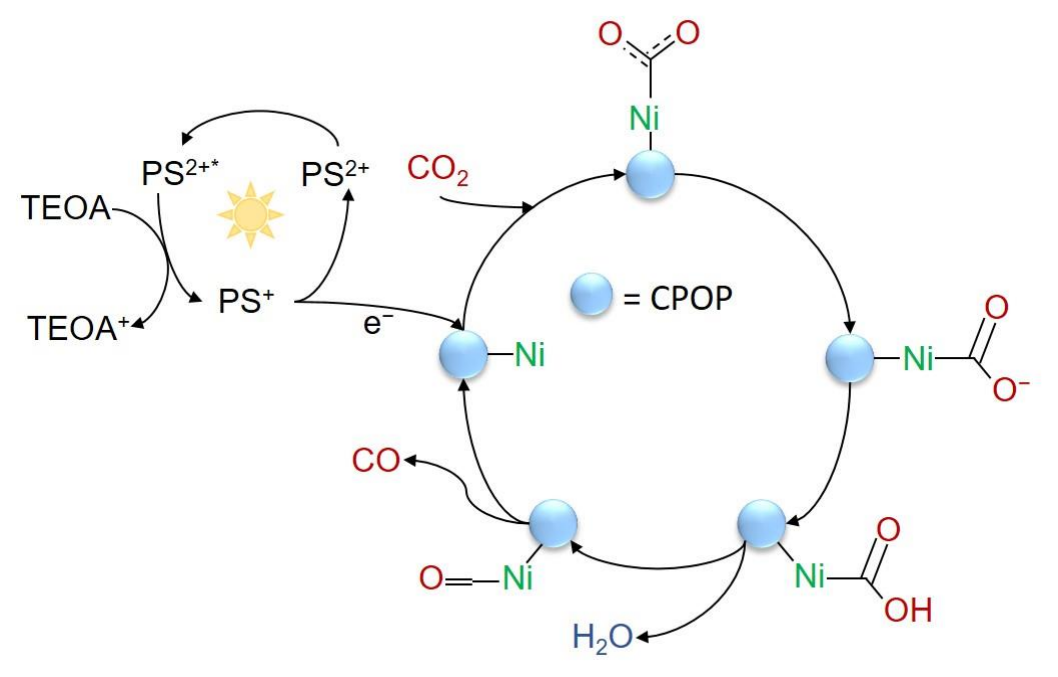
Proposed mechanism for the photocatalytic CO_2_ reduction by a Ni-loaded CPOP. Data from Ref. [75].

Further networks such as porous polycarbazoles or porous covalent triazine frameworks were investigated as materials to support photocatalytic carbon dioxide reduction. Just recently in 2022, Byun and Wang published their work on crystalline porous organic polymers (CPOPs) consisting of polycarbazoles functionalized with various transition-metal complexes. The CPOPs were synthesized by simple one-pot oxidative coupling polymerization by using 1,3,5-tri(9H-carbazol-9-yl)benzene as a precursor, promoted by FeCl_3_. CPOPs themselves, showed a capacity to capture CO_2_ due to their microporosity and a high surface area. After synthesis, the obtained CPOPs were dispersed in chloroform and mixed with either iron chloride, cobalt chloride, or nickel chloride for metal impregnation to give CPOP-Fe, CPOP-Co, and CPOP-Ni, respectively. The metal species were anchored within the porous network, as proven by scanning electron microscopy, FT-IR, powder-XRD, N_2_ physisorption, and diffuse reflectance (DR) measurements. The unpaired electrons in the 3d-orbitals of the transition metal centers were delocalized and were able to react with anti-bonding π-orbitals of CO_2_. Photocatalytic activity was tested in aqueous acetonitrile solution under visible light irradiation with [Ru(bpy)_3_]^2+^ as a photosensitizer and TEOA as a sacrificial agent. The mechanism for a Ni-loaded CPOP was proposed as follows: PS^2+^ was excited by light absorption and PS^2+*^ was formed. TEOA reduced this state to give PS^+^. The excited electron was transferred to the Ni active site at which CO_2_ was coordinated to the metal. Activation of the C=O double bond led to negatively charged CO_2_^−^. The electron was transferred to the adsorbed CO_2_ and formed a CO_2_* intermediate. After further protonation and reduction, H_2_O and CO were released (Figure 11). Different metal ions had an impact on the kinetics of activation and, consequently, product selectivity and production rate. The CPOP-Co showed the best results, with a CO production rate of 31,000 μmol g^−1^ h^−1^; however, this just led to a CO selectivity of 55%. While CPOP-Ni produced CO with a production rate of 28,000 μmol g^−1^ h^−1^ with 89% selectivity, CPOP-Fe lacked in both production rate and selectivity compared to the other two transition metals [75].

Huang and Cao were the first to use porous covalent triazine frameworks (CTFs), modified with a rhenium-based catalyst, for photocatalytic CO_2_ conversion [76]. CTFs had already been known as gas adsorption materials due to their porous structure [76,77,78]. The CTF structure was based on 2,6-dicyanopyridin (CTF-py), so that the nitrogen units provided the possibility to act as anchor sites for metal catalyst molecules. A [Re(CO)_5_Cl] complex, which can act as both photosensitizer and photocatalyst, was coordinated to these nitrogen sites via ionic thermal synthesis to give Re-CTF-py networks. In a gas–solid system including TEOA as a sacrificing agent, CO was produced with a rate of up to 353 μmol g^−1^ h^−1^ and a TON of 4.8 within 10 h. In this solid-gas system, the photocatalyst was not dissolved in a solution, instead the Re-CTF-py was dispersed on a quartz film and a MeCN/water mixture was added to the cell. Leaching of the active Re sites was prevented and the catalyst showed better recyclability [76].

**Figure 12:**
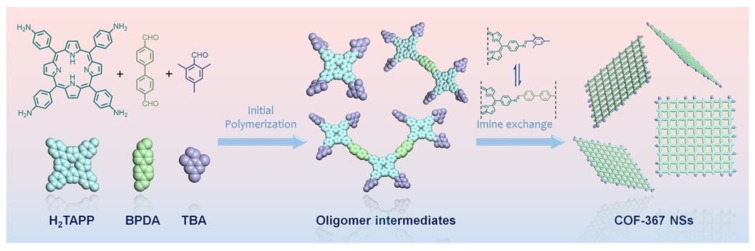
Schematic illustration of the synthesis of COF-367 nanosheets (NSs) via an imine-exchange strategy. Using CoTAPP instead of H_2_TAPP led to CoPor based COF-367-Co NSs. Reprinted with permission from Ref. [79]. Copyright 2019, American Chemical Society.

Wang, Zeng, and Jiang opened the path for a scalable bottom-up synthesis of two-dimensional (2D) COF nanosheets. The synthesis approach included an imine-exchange strategy. 5,10,15,20-tetra(*p*-aminophenyl)porphyrin (H_2_TAPP) reacted with 4,4′-biphenyldialdehyde (BPDA) under solvothermal conditions. Afterwards, the addition of an excess amount of 2,4,6-trimethylbenzaldehyde (TBA) enabled the 2D imine-linkage to give COF-367 nanosheets (NSs) (Figure 12). TBA prevented π-π stacking of the single COF nanosheets due to methyl side groups, enabling anisotropic growth along the planar nanosheet direction. Using CoTAPP instead of H_2_TAPP resulted in Co(II) porphyrin (CoPor)-based COF-367-Co NSs. As a proof-of-concept, the photocatalytic performance of the COF-367-Co NSs was explored under visible light irradiation. In an 0.1 M KHCO_3_ aqueous solution with [Ru(bpy)_3_]^2+^ photosensitizer and ascorbic acid (AA) as the sacrificial agent and electron donor, the production rate of CO from CO_2_ was remarkably high, with a value of 10,162 μmol g^−1^ h^−1^ and a CO selectivity of 78% in comparison to H_2_. The whole COF-367-Co catalytic system remained almost stable with a production rate of still 10,000 μmol g^−1^ h^−1^ after six successive cycles. The bulk material, not having a 2D-layered structure, exhibited a CO production rate of only 124 μmol g^−1^ h^−1^. Mechanistic investigations were performed using photoluminescence and ultrafast transient absorption spectroscopic experiments. These experiments revealed that [Ru(bpy)_3_]^2+^ became excited, while AA sacrificed one electron to the Ru to achieve [Ru(bpy)_3_]^+^. This electron was further transferred from the Ru complex to the COF. Within the COF, CO_2_ was coordinated to the Co(II) atoms. With the electron transfer, COOH* was formed as the coordinated intermediate species. This formation turned out to be the reaction limiting step. DFT calculations revealed a small energy barrier of 0.47 eV for the intermediate formation process. The small energy barrier facilitated the process to occur at room temperature. These insights uncovered that the 2D nanosheet structure enhanced the photocatalytic performance because of its large aspect-ratio. The 2D morphology caused a large number of active sites for a high adsorption capacity on the surface. Compared to the respective bulk material, in which the active sites are rather hidden inside the bulk, the accessibility of active sites on the surface of the nanosheets increased the rate of CO_2_ reduction [79].

#### 3.2.2. Amorphous POPs

**Figure 13:**
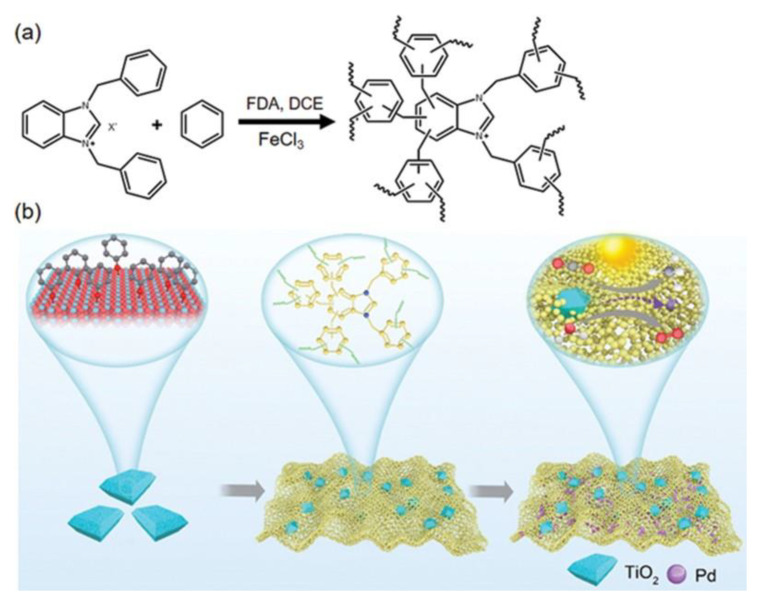
(**a**) Synthesis of NHC-containing HCPs; (**b**) Scheme of the in situ knitting strategy to form HCP-TiO_2_-Pd. Reprinted with permission from Ref. [83]. Copyright 2021, Wiley-VCH.

In 2019, Tan et al. stated that high CO_2_ conversions needed both efficient photocatalysis with high CO_2_ uptakes and short diffusion paths. Therefore, they reported on a novel material which consisted of a porous hyper-crosslinked polymer on TiO_2_ functionalized graphene (HCP-TiO_2_-FG). TiO_2_ is a low cost photocatalyst, HCPs provided a porous network. By the linkage, the advantages of all materials were combined. The material was synthesized by a synthetic strategy which included in situ knitting [80]. First, the functionalized TiO_2_-Graphene (TiO_2_-FG) was manufactured from TiO_2_-graphene (TiO_2_-G) by a solvothermal process and the hydroxyl groups on the TiO_2_ surface were converted to phenyl groups [81]. The polymer layer was hyper-crosslinked on this TiO_2_-FG skeletal structure. Polymer layers were hyper-crosslinked on the TiO_2_-FG by knitting of the phenyl groups and 1,3,5-Triphenylbenzene (*syn-*PhPh_3_), resulting in HCP-TiO_2_ [82]. Due to the porosity, a high surface area of 988 m^2^g^−1^ and a CO_2_ uptake efficiency of 12.87 wt.% were measured. Without the use of sacrificial agents or a co-catalyst, CH_4_ production via a multi-electron process in a gaseous set-up reached a value of 27.63 μmol g^−1^ h^−1^ and a CH_4_ selectivity of 83.7% was measured. Methane is a higher valued product than CO as CH_4_ can be used as a raw product to be transformed into more complex organic molecules. The composite structure enables a high CO_2_ uptake in its pores, as well as short diffusion paths between adsorption and catalysis. Moreover, the HCP absorbed light in a broad range and the graphene layer improved charge separation due to fast charge mobilities [80]. In a similar approach, the same group used HCPs grafted onto a TiO_2_ surface metalized with Pd (HCP-TiO_2_-Pd). Pd nanoparticles could capture photogenerated electrons and worked as an electron trap. This approach improved the charge separation for the complete reduction reaction. The HCPs were synthesized by copolymerizing N-heterocyclic carbenes (NHC) and benzene. The HCP contained N-heteroatom sites. These were induced defects acting as anchor points for Pd nanoparticles which performed as a co-catalyst. Friedel-Crafts reaction was used to link the phenyl groups of the TiO_2_ surface with the HCP network. Because the heteroatom-induced defects of the HCP were spread over the whole material, the Pd sites were distributed evenly (Figure 13). Extensive morphology investigation including high-angle annular dark-field (HAADF) and energy-dispersive spectroscopy (EDS) mapping images proved the covalent linkages, building of a porous HCP network and uniform dispersion of active sites. The photocatalytic performance was tested in a gas–solid reaction system under visible light irradiation, but without the addition of further sacrificial agents or photosensitizers. HCP-TiO_2_-Pd showed a production rate of CH_4_ of 237 μmol g^−1^ h^−1^ and a selectivity of more than 99.9%. Structural stability was also given; the production rate was maintained with a value of over 80%, even after five cycles. Pd favored subsequent hydrogenation reduction in CO_2_, enabling methane formation with a high selectivity [83].

**Figure 14:**
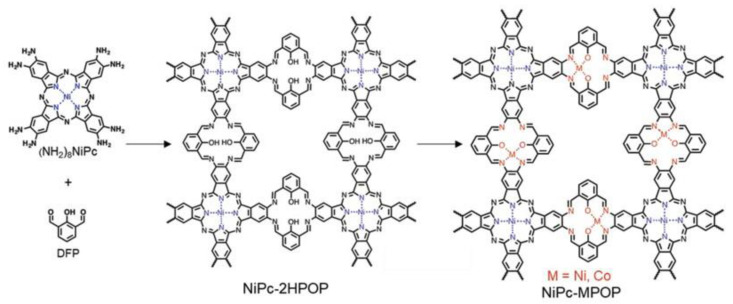
Scheme of the stepwise synthesis of NiPc-MPOP (M = Ni or Co). Reprinted with permission from Ref. [84]. Copyright 2021, Wiley-VCH.

Wang and Zang integrated catalysts to POPs with two different environments, which were metallophthalocyanine and salphen moieties [84]. Metallophthalocyanine POPs with M-N_4_ (M = Co, Ni, Cu or Fe) sites are already widely known as active materials for electro- and photocatalysis of CO_2_ [73,85,86]. Besides the M-N_4_ metallophthalocyanine environment with M = Ni, salphen M-N_2_O_2_ (M = Ni, Co) moieties were integrated in the materials as a second environment. The material was synthesized by polycondensation reaction of nickel(II)-2,3,9,10,16,17,23,24-octakis(amino)phthalocyanine ((NH_2_)_8_NiPc) with 2,6-diformylphenol (DFP) in a dimethylacetamide/mesitylene mixture and with the regulating agent aniline. This step was followed by treatment with Ni(OAc)_2_ × 4 H_2_O or Co(OAc)_2_ × 4 H_2_O, resulting in a porous framework with atomically isolated Ni-N_4_ and M-N_2_O_2_ (M = Ni, Co) sites (Figure 14), denoted as NiPc-NiPOP or NiPc-CoPOP (Figure 14). FT-IR spectroscopy, XPS, Fourier transformed extended X-ray absorption fine structure (FT-EXAFS) spectroscopy, high-angle annular dark-field scanning transmission electron microscopy (HAADF-STEM) and EDS mapping revealed an amorphous structure of the samples and confirmed that one metal ion was incorporated in each of the salphen pockets. The BET surface area was determined to be 333 m^2^ g^−1^. Nonlocal density functional theory (NLDFT) proved the mesoporous character and the accessibility of the catalytic sites. Photocatalytic experiments were conducted under visible light in acetonitrile/water solution with additional [Ru(bpy)_3_]^2+^ photosensitizer and TEOA sacrificial agent and monitored by gas chromatography. The NiPc-NiPOP material had an extraordinary CO generation of 7.77 mmol g^−1^ with a CO selectivity of 96% after four hours of reaction time and performed better than NiPc-CoPOP. Control experiments and theoretical DFT studies showed that the salphen Ni-N_2_O_2_ units were more active for CO_2_ reduction than the metallophthalocyanine sites [84].

### 3.3. Metal-Organic Frameworks

Metal–organic frameworks (MOFs) are hybrid crystalline materials, also known as porous coordination polymers (PCPs) or porous coordination networks (PCNs) [87]. They are built by metal ions or metal clusters, serving as nodal points, that are coordinatively linked by organic ligands [88]. The combination of different metals and organic linkers opens the path to millions of distinct MOFs, i.e., over 500,000 have been predicted and 90,000 synthesized so far [89]. Therefore, the number of MOFs reported regarding CO_2_ reduction studies is also enormously high. We recommend comprehensive reviews [90,91] on MOFs for carbon dioxide photocatalytic reduction.

MOFs can build 1D, 2D and 3D structures. Among the structural diversity, the main MOF systems that have been investigated so far are zeolitic imidazolate frameworks (ZIFs), carboxylate MOFs, and zirconium-based MOFs [92]. The properties of a MOF are determined by the network connectivity of the single molecules. MOFs have a large internal surface area, and their pores have controllable sizes which have adsorptive sites for capturing gas molecules [14,93]. Tailored MOFs offer the opportunity of incorporating functional species, e.g., in the form of nanoparticles, within the frameworks, which can be prepared within the cavities or be directly encapsulated in the MOFs [94]. Tunable pore sizes as well as surface areas and chemical composition make MOFs versatile materials for photocatalytic CO_2_ conversion [95,96]. In a MOF, the metal clusters can serve as reductive sites for CO_2_ reduction, while the organic linkers enhance CO_2_ adsorption and charge transport [87,91].

In 2014, Luo et al. used iridium as an active light-harvesting agent due to its broad light absorption range and the longevity of its excited states. Ir(III) was embedded into a bifunctional iridium-yttrium coordination polymer photocatalyst of Y[Ir(ppy)_2_(dcbpy)]_2_[OH] (ppy = 2-phenylpyridine, dcbpy = 2,2′-bipyridine-4,4′-dicarboxylate). The generated complex acted as a photosensitizer for harvesting visible light and, at the same time, as a catalyst for CO_2_ reduction. The [Y(OH)_2_(CO_2_)_2_]∞ chains were constructed by Y^3+^ ions acting as multi-coordination centers bridged by two OH^-^ units and two carboxylate groups. This coordination enhanced the stability to construct a 3D supramolecular framework. CO_2_ was reduced to HCOO^−^ in a mixture of MeCN and TEOA with a TOF of 119 μmol g^−1^ h^−1^. The authors proposed a mechanism for MOF-stabilized photocatalyst-mediated CO_2_ reduction as follows. After the catalyst was excited by visible light irradiation, TEOA reduced this state as a sacrificial agent. When two adjacent excited units underwent a one-electron transition and transferred an electron to CO_2_, carbon dioxide was reduced to HCOO^−^ in a two-electron process [97].

**Figure 15:**
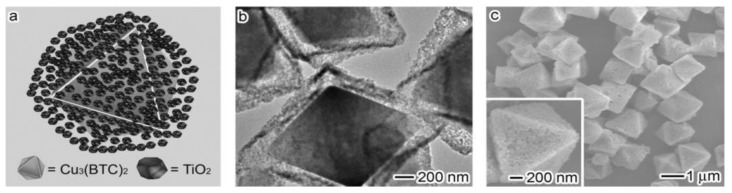
(**a**) Illustration of the core–shell structure; (**b**) TEM image; (**c**) SEM image of Cu_3_(BTC)_2_@TiO_2_ structure. Reprinted with permission from Ref. [98]. Copyright 2014, Wiley-VCH.

Jiang, Zhang, and Xiong implemented MOFs in a photocatalysis for gaseous reactions under UV light irradiation in 2014. For this purpose, the catalyst was not dissolved in an aqueous media and only interacted in its solid form with gaseous molecules. Effective molecular adsorption and activation were crucial for such a system. The authors developed a hybrid MOF-metal core–shell structure, as proven by TEM and SEM images. The microporous core of the MOF was built by Cu_3_(BTC)_2_ (BTC = benzene-1,3,5-tricarboxylate) and a semiconductor (SC) TiO_2_ was included as a macroporous shell for generating excitons (Figure 15). The MOF-TiO_2_ structure was referred to as Cu_3_(BTC)_2_@TiO_2_. CO_2_ was able to penetrate the macroporous shell structure and was captured in the microporous MOF core in which it was adsorbed on the Cu sites. The mechanism was investigated via ultrafast transient absorption. When TiO_2_ was photoexcited, electron-hole pairs were formed. The electron was transferred to the Cu_3_(BTC)_2_ MOF structure including the catalytic metal atoms. Upon receiving the electrons, Cu was activated, and CO_2_ reduction of adsorbed molecules took place via an eight-electron process, resulting in CH_4_. The CH_4_ production rate of Cu_3_(BTC)_2_@TiO_2_ was determined as 2.64 μmol g^−1^ h^−1^, whereas bare TiO_2_ produced 0.52 μmol g^−1^ h^−1^ CH_4_ and 2.29 μmol g^−1^ h^−1^ H_2_. The MOF structure and SC improved the charge transfer from the SC to MOF and thus the charge separation within the SC [98].

Recent publications indicated that solely superficial layers on surfaces were involved in photoreactions due to restricted diffusion barriers [99,100,101,102]. Consequently, a new class of material has emerged which are 2D metal-organic layers (MOLs). MOLs are basically a monolayer version of MOFs, also referred to as MOF nanosheets.

**Figure 16:**
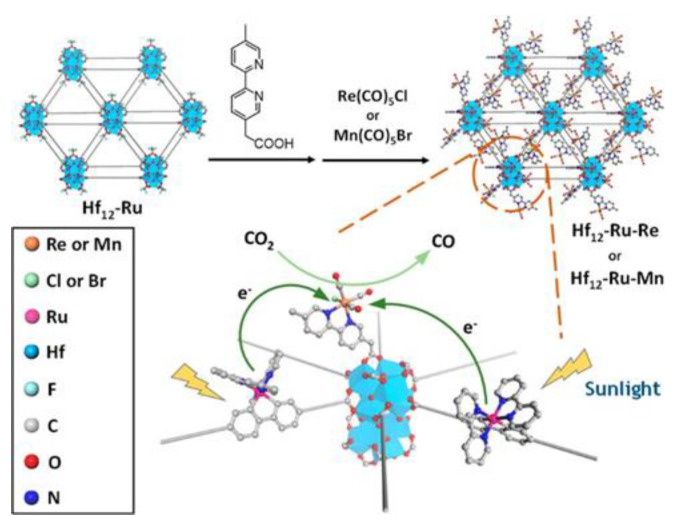
Schematic synthesis of Hf12-Ru-Mn and Hf12-Ru-Re and proposed sunlight-driven mechanism. Reprinted with permission from Ref. [103]. Copyright 2018, American Chemical Society.

In 2018, Lin et al. published the first photosensitizing MOL made from secondary Hf_12_ building units and [Ru(bpy)_3_]^2+^-derived dicarboxylate ligands by solvothermal reaction. Carboxylate exchange reactions enabled a modification of the Hf_12_-Ru surface with an additional rhenium(I) or manganese(I) catalyst (Figure 16). The monolayer structure of 1.6 nm thickness was analyzed via HR-TEM, XRD and AFM measurements among others. The materials Hf_12_-Ru-Mn or Hf_12_-Ru-Re possessed both functionalities of a photosensitizer and metal catalyst. CO_2_ photoreduction was carried out in acetonitrile after the addition of TEOA, and BNAH or 1,3-dimethyl-2-phenyl-2,3-dihydro-1H-benzo[d]imidazole (BIH) as an electron donor. Hf_12_-Ru-Re together with BIH showed a product selectivity for CO of 98% and a TON of up to 3849 in 24 h under artificial light irradiation. Using BNAH as sacrificial electron donor the TON reached 2092 in 24 h. Remarkably, CO_2_ reduction under sunlight radiation was feasible with the Hf_12_-Ru-Re MOL and BIH and could reach a TON of 670 within 6 h of irradiation. Photophysical and electrochemical mechanism studies included methods such as phosphorescence spectroscopy, CV and differential pulse voltammetry (DPV). It revealed that the BIH reduced Hf_12_-Ru units, acting as photosensitizing centers, which were then negatively charged enough to reduce the catalytic rhenium units (Figure 16) [103].

Peng et al. reported on MOLs made from Ni_3_(HITP)_2_ (HITP = 2,3,6,7,10,11-hexaiminotriphenylene) with a planar Ni-N_4_ coordination motif [104]. The material had been known as a semiconducting metal-organic graphene analogue with a high conductivity for charge transportation [105]. The height profile measured by AFM microscopy revealed a thickness of 4.2 nm which showed that the nanosheet was made out of only few layers of 2D MOFs. For CO_2_ reduction, the MOL performed as a co-catalyst in hybrid catalytic systems under visible light irradiation with [Ru(bpy)_3_]^2+^ as photosensitizer and TEOA as sacrificial agent. In a solvent mixture of water and acetonitrile, CO was formed with a selectivity of 97% and a production rate of 3450 μmol g^−1^ h^−1^. Even after six cycles, the material proved to be stable [104]. The photocatalytic activity of Ni_3_(HITP)_2_ was further improved by adding reduced graphene oxide (rGO) that interacted with the catalyst via Coulomb interactions. The heterostructure had an impact on the electronic structure of the metal centers due to electrostatic charge transfer and π-π interactions. The CO production rate was improved to up to 3800 μmol g^−1^ h^−1^ with 92% CO selectivity. PL and PL decay, as well as ultraviolet photoelectron spectroscopy (UPS), accompanied by DFT calculations, suggested a division of the mechanistic process into three main steps: (1) photon absorbance of the photosensitizer and thus being transformed to the excited state, (2) the photosensitizer transferred its excited electron to the Ni_3_(HITP)_2_ located in the nanosheet layer, (3) CO_2_ was reduced to CO at the Ni-N_4_ sites. In detail, the photoexcited electron in the [Ru(bpy)_3_]^2+*^ was delocalized in the π* orbital. The formed hole was neutralized by reduction from the sacrificial electron donor TEOA. The electron was transported within the conduction band of the nanosheet to the Ni-N_4_ sites caused by the metallicity of the MOL. Ni-N_4_ sites acted as catalysts due to their enhanced electron density. Electrophilic adsorption of CO_2_ and H^+^ on catalytic Ni-N_4_ sites was promoted. COOH* was formed as an adsorbed intermediate on the catalytic centers. Protonation of the intermediate plus reduction released H_2_O and CO. The rGO enhanced the electrophilicity of Ni-N_4_ sites and performed as an electron relay of the photogenerated electrons to the conduction band of the metal centers for reduction [106].

**Figure 17:**
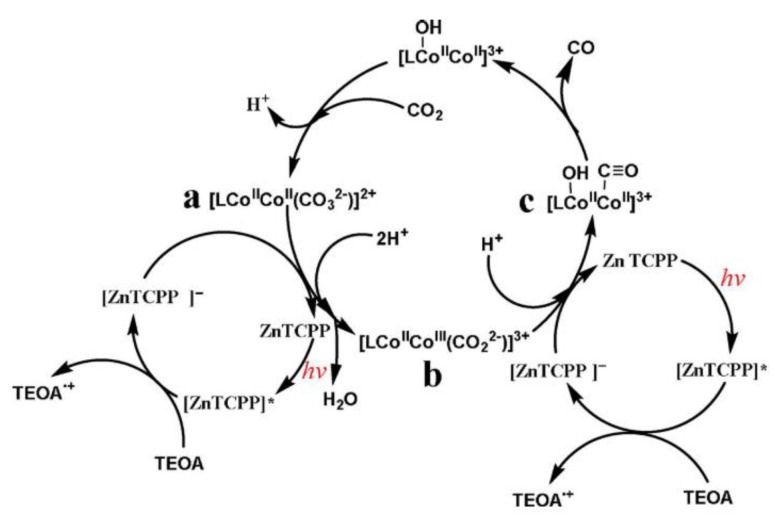
Proposed mechanism for the photocatalytic CO_2_ reduction to CO with a Zn-MOL and [Co_2_[(OH)L](ClO_4_)_3_ as co-catalyst. Reprinted with permission from Ref. [107]. Copyright 2018, Elsevier B.V.

Gao et al. published new noble-metal-free hybrid photocatalytic nanosheets with 2D zinc(II) porphyrin-based networks of 5,10,15,20-tetrakis(4-carboxyphenyl) porphyrin (TCPP), called Zn-MOF NSs or Zn-MOLs. Photocatalytic reduction was utilized with a binuclear cobalt complex, namely [Co_2_[(OH)L](ClO_4_)_3_ (L = N[(CH_2_)_2_NHCH_2_(m-C_6_H_4_)CH_2_NH(CH_2_)]_3_N), as co-catalyst, besides TEOA as an electron donor. AFM measurements revealed a thickness of 4.7 nm of the NSs. The photocatalytic system including the co-catalyst showed a high CO selectivity of 91% and a TON of 118, which was almost twice the value compared to the bulk material of the respective Zn-MOF (TON_CO_ = 64). The Zn-MOLs proved also to be stable under visible light irradiation. The 2D network had a higher charge transport ability and thus an enhanced charge separation compared to the bulk material [107]. Based on findings from Lu et al. [108], Gao et al. proposed a mechanism for the CO_2_ photoreduction to CO with Zn-MOLs (Figure 17). The building block of the Zn-MOL nanosheets, which is ZnTCPP, formed [ZnTCPP]* after being photoexcited. TEOA reduced this intermediate to [ZnTCPP]^-^. CO_2_ was coordinated to the binuclear cobalt complex containing Co_2_^II,II^ which formed a carbonate-bridged complex. Via proton-coupled electron transfer with the help of [ZnTCPP]^-^, complex b was formed containing two Co centers with two different oxidation states (Co_2_^II,III^). After another proton-coupled electron transfer with [ZnTCPP]^-^, c was formed containing two Co^II^ centers. Finally, CO was released, and the binuclear cobalt complex can be reintroduced in the catalytic cycle [107].

## 4. Conclusions

This review summarizes the recent developments in photocatalytic CO_2_ conversion using metal-containing polymers. New materials and mechanistic insights were revealed for different material classes, such as supramolecular and coordination polymers, porous organic polymers, and porous coordination polymers (MOFs and MOLs). Table 2 presents an overview of the key reports mentioned in this review.

1D-coordination polymers were revealed to be a simple material for CO_2_ reduction. Even systems without the necessity of an additional photosensitizer were already published. Moreover, supramolecular and coordination polymers are a class of efficient supports for photocatalysts. Amphiphilicity of the systems helped to prevent the degradation of the catalytic centers in an aqueous system. Additionally, self-assembled materials and hydrogels facilitated simple co-location of photosensitizer and catalysts. Proximities of the catalytic metal centers enhanced intramolecular charge transfers, but mechanistic details on new materials are still missing. Porous organic polymers that were synthesized as 3D frameworks or 2D layers performed as a versatile class of materials due to their porosity. Their tunable characteristics such as light absorption ability and energies of the band gaps were used for improvements concerning selectivity and stabilities. The field of POPs supported by non-noble metals or inorganic semiconductors, especially, was recently broadened and complemented by unraveling mechanistic details. Metal-organic frameworks have shown to have accessible pores for small molecules and can additionally already carry photocatalytically active metal centers in their basic structure. Research on this enormous variety of structures has shown that the composition of the whole system and the choice of solvent have a strong impact on carbon dioxide adsorption ability, product selectivity, catalyst stability, productions rates, etc. In general, high electron densities and improved electron-hole separation support CH_4_ formation over carbon monoxide generation. However, since both products are still C1 building blocks, photocatalyst efficiencies have to be pushed to the next level for C2 product formation.

Most of the presented photocatalytic performances have been executed in saturated CO_2_ atmospheres and watery systems on a lab scale. For translation of these systems to an industrial-relevant production scale, some drawbacks need to be improved. One drawback is that the flue gas for conversion is often not pure CO_2_ gas. Other gases, such as N_2_, NO_2_, can also be contained in the gas streams. The selectivity for CO_2_ adsorption in the presence of other gas molecules on the catalytic metal centers needs to be maximized. Moreover, gas streams have rather low pressures. Efficient adsorption of carbon dioxide on catalytic active sides under low CO_2_ pressure and concentrations must be facilitated for generating an industrial relevance of photocatalytic carbon dioxide reduction. As a consequence, sophisticated processes are so far only performed under laboratory utilization and need to be translated to industrial scales.

## Figures and Tables

**Table 1:** Half-cell reactions and potentials vs. the NHE at pH = 7 [33,34].

Half-Cell Reaction	*E*^0’^ in V
2 H_2_O → O_2_ + 4 H^+^ + 4 e^−^	+0.82
2 H^+^ + 2 e^−^ → H_2_	−0.41
CO_2_ + 2 H^+^ + 2 e^−^ → CO + H_2_O	−0.52
CO_2_ + 2 H^+^ + 2 e^−^ → HCOOH	−0.61
CO_2_ + 4 H^+^ + 4 e^−^ → C + 2 H_2_O	−0.20
CO_2_ + 4 H^+^ + 4 e^−^ → HCHO + H_2_O	−0.48
CO_2_ + 6 H^+^ + 6 e^−^ → CH_3_OH + H_2_O	−0.38
CO_2_ + 8 H^+^ + 8 e^−^ → CH_4_ + 2 H_2_O	−0.24

**Table 2:** Overview of key reports including used catalyst system, solvent, light source and the main product after photocatalytic CO_2_ reduction.

Catalyst Support	Photocatalytic System	Solvent	Light Source	Main Products	Ref.
Triblock amphiphilic micelles	Re (Cat, PS), TEOA (SA)	H_2_O	500 W Xe (λ ≥ 400 nm)	CO, H_2_	[42]
Poly(vinyl bipyridine)	Re (Cat), Ru (PS), TEOA+BIH (SA)	DMF	green LED (λ = 520 ± 30 nm)	CO	[44]
Polymers on quantum dots	CdS-Ni assembly (Cat, PS), TEOA (SA)	H_2_O	LED (λ = 420 nm)	CO, H_2_	[48]
Ullazine supramolecular polymers	Co (Cat), Chromophores (PS), TEOA (SA)	MeCN/H_2_O	blue LED (λ = 450 nm)	CO, CH_4_	[52]
Metal-organic coordination polymer gel	Ru (Cat), Porphyrin (PS), TEA/BNAH (SA)	MeCN/H_2_O	300 W Xe lamp (λ > 400 nm)	CO, CH_4_	[54]
Bipyridine COF	Re (Cat), Ir (PS), TEOA (SA)	MeCN	300 W Xe (λ > 420 nm cut-off filter)	CO, H_2_	[69]
PMO with bipyridyl ligands	Ru (Cat), Ru (PS), BNAH (SA)	DMA/H_2_O	500 W Hg (λ > 430 nm)	CO, HCOO^−^	[71]
Porous CTF	Re (Cat, PS), TEOA	none	300 W Xe (λ = 200–1100 nm)	CO	[76]
HCP-TiO2-Pd	TiO2 (Cat), Pd (co-Cat)	none	300 W Xe, UV-vis light	CH_4_	[83]
Metallophthalocyanine and salen POPs	Ni (Cat), Ru (PS), TEOA (SA)	MeCN/H_2_O	white LED (λ = 400–800 nm)	CO	[84]
Hybrid MOF-metal core–shell structure	Cu (Cat), TiO_2_ (PS)	none	300 W Xe (λ < 400 nm)	CH_4_	[98]
MOLs made from Ni_3_(HITP)_2_	Ni (Cat), MOL (co-Cat), Ru (PS), TEOA (SA)	TEOA/H_2_O/MeCN	100 W LED (λ = 420 nm)	CO	[104]
